# Association Between Body Mass Index and All-Cause Death in Japanese Population: Pooled Individual Participant Data Analysis of 13 Cohort Studies

**DOI:** 10.2188/jea.JE20180124

**Published:** 2019-12-05

**Authors:** Atsushi Hozawa, Takumi Hirata, Hiroshi Yatsuya, Yoshitaka Murakami, Shinichi Kuriyama, Ichiro Tsuji, Daisuke Sugiyama, Atsushi Satoh, Sachiko Tanaka-Mizuno, Katsuyuki Miura, Hirotsugu Ueshima, Tomonori Okamura

**Affiliations:** 1 Department of Preventive Medicine and Epidemiology, Tohoku Medical Megabank Organization, Sendai, Japan; 2 Department of Public Health, Fujita Health University School of Medicine, Toyoake, Aichi, Japan; 3 Department of Medical Statistics, School of Medicine, Toho University, Tokyo, Japan; 4 Department of Disaster Public Health, International Research Institute of Disaster Science, Tohoku University, Sendai, Japan; 5 Division of Epidemiology, Department of Health Informatics and Public Health, Tohoku University School of Public Health, Graduate School of Medicine, Sendai, Japan; 6 Department of Preventive Medicine and Public Health, Keio University School of Medicine, Tokyo, Japan; 7 Department of Preventive Medicine and Public Health, Faculty of Medicine, Fukuoka University, Fukuoka, Japan; 8 Department of Medical Statistics, Shiga University of Medical Science, Otsu, Japan; 9 Department of Public Health, Shiga University of Medical Science, Otsu, Japan; 10 Center for Epidemiologic Research in Asia, Shiga University of Medical Science, Otsu, Japan

**Keywords:** body mass index, pooled analyses, all-cause mortality, prospective studies

## Abstract

**Background:**

We sought to investigate the optimal values of BMI for the lowest risk of all-cause death and whether the optimal BMI differs according to smoking status in large-scale pooled analysis of 13 Japanese cohorts.

**Methods:**

Data from 179,987 participants of 13 well-qualified cohort studies conducted throughout Japan were used for our analysis. A cohort-stratified Cox proportional hazard model was used. *P* values for interactions were calculated based on the cross product of BMI and age, sex, or smoking status.

**Results:**

In the entire study population, all-cause mortality risk was lowest when the BMI was 22.0–24.9 kg/m^2^. This was also the case for selected healthy participants (never smoked, baseline total cholesterol level ≥4.1 mmol/L; the first 5 years of follow-up data were excluded). No effect modification of age, sex, or smoking status was observed. Regardless of their BMI, never smokers always had a lower all-cause mortality risk than did current smokers even with an ideal BMI in terms of mortality risk.

**Conclusion:**

A BMI of 22–24.9 kg/m^2^ correlated with the lowest risk of mortality, regardless of whether all participants or selected healthy participants were analyzed. The fact that smoking was more strongly associated with mortality than obesity emphasizes the urgency for effective anti-smoking programs.

## INTRODUCTION

The U-shaped or J-shaped association between body mass index (BMI) and all-cause death was shown in previous studies.^1^^–^^10^ Among individuals with low BMI values, smoking status is one of the major confounding factors of the association between BMI and all-cause death.^11^^–^^15^ To identify the optimal BMI is important to set the target value for preventing all-cause death. However, the optimal values of BMI for the lowest risk of all-cause death are unclear. In addition, whether the optimal BMI differs according to smoking status in Japanese was uncertain. If the optimal BMI differs, we may change the target value of the BMI according to smoking status. Meanwhile, it is not easy to detect the precise association between BMI and all-cause death because the association is generally influenced by various confounders. Thus, we need to analyze large-scale data stratified by some confounders. We believe that our large cohort database makes it possible to answer our clinical questions.

The Evidence for Cardiovascular Prevention from Observational Cohorts in Japan (EPOCH-JAPAN) study analyzed pooled data from multiple investigations of the relation between health examination measures, such as laboratory measures and lifestyle factors, and mortality in the Japanese population.^16^^–^^19^ We aimed to determine the optimal BMI in relation to the lowest risk of all-cause death in the Japanese population using a large-scale pooled analysis of Japanese cohort studies. We also investigated whether optimal BMI differs according to smoking status.

## METHODS

### Study participants

The EPOCH-JAPAN study was a pooled analysis of 13 well-qualified cohort studies (Tanno-Sobetsu study, Ohsaki cohort study, Ohasama study, Oyabe study, Yoshida Kogyo Kabushikigaisha [YKK] workers study, Suita study, Radiation effects research foundation [RERF] cohort study, Hiyasama study, Japan collaborative cohort [JACC] study, National Integrated Project for Prospective Observation of Non-communicable Disease And its Trends in the Aged [NIPPON DATA]80, NIPPON DATA90, Ibaraki Prefectural Health Study [IPHS], and Shiga Prefectural Medical Insurance [SPMI] cohort study) conducted in Japan.^16^^–^^19^ It included a total of 188,321 participants (70,613 men and 117,708 women) aged 40–89 years at baseline. The baseline years were from 1987 to 1995, and the average follow-up period was 10 years. The details of this project have been described previously.^16^ All cohort studies contributing to the EPOCH-JAPAN study were approved by the research ethics committees at each study center and have been described in detail in peer-reviewed publications.

A total of 187,292 participants aged 40–89 years who measured BMI at baseline from 13 cohorts were included in the present study. We excluded 7,305 participants owing to missing data for smoking status (*n* = 4,798) or total cholesterol (TC) (*n* = 2,507). Finally, 179,987 participants (68,282 men and 111,705 women) from 13 cohorts were included in our analysis.

### Measurements

The study endpoint was all-cause death, and the exposure of interest was BMI. Body weight in light clothing was measured using a standardized body weight calculator, and BMI was calculated based on the height in stocking feet and the weight. We set age, smoking status, and TC level as covariates. We classified smoking status into non-smoker, past-smoker, and current smoker. In two cohorts (Ohasama and Oyabe), former smokers were classified as non-smoker. TC level was measured using enzymatic method in all cohorts.

### Statistical analysis

Continuous variables are shown as the mean and standard deviation or the median and interquartile range. Categorical variables are shown as the number and proportion. We calculated the crude mortality rate and the hazard ratio (HR) for all-cause death.

To describe the all-cause mortality risk associated with a range of BMIs, we equally divided the participants into 40 groups according to its distribution. We estimated age- and smoking-adjusted HRs and 95% confidence intervals based on an arbitrarily determined reference category (the 24th quantile: mean BMI, 23.8 kg/m^2^). A Cox proportional hazard model was used, and the study cohorts were treated as stratification variables.^16^

We analyzed the relation between BMI and all-cause mortality in a sample restricted to healthy participants who had never smoked and had a TC level ≥4.1 mmol/L (160 mg/dL) at baseline to exclude the effects of smoking, malnutrition, and exhaustion on BMI.^4^ This sub-group analysis did not include the data from the first 5 years of the follow-up period.^4^

To assess interactions, we used 11 BMI categories: <19, 19–19.9, 20–20.9, 21–21.9, 22–24.9 (reference category), 25–25.9, 26–26.9, 27–27.9, 28–28.9, 29–29.9, and ≥30 kg/m^2^. To examine multiplicative interactions between age and BMI, we determined the significance of the cross product of each age group (40–64, 65–74, and 75–89 years) and BMI category using the likelihood ratio test. A similar method was used to examine interactions between sex and BMI and smoking status (excluding past smokers) and BMI.

To determine the association between BMI and all-cause mortality according to age, sex, and smoking status, we used a combination of the age, sex, smoking status, and BMI categories. In these analyses, 40–64 year-old men who had never smoked and had a BMI of 22–24.9 kg/m^2^ were used as the reference group.

All statistical analyses were performed using SAS version 9.4 software (SAS Institute Inc., Cary, NC, USA).

## RESULTS

Our study included 179,987 (68,282 men and 111,705 women) participants. The baseline characteristics of study participants according to BMI category are shown in Table 1. The mean age of all participants was 58.7 years, and the mean BMI was 23.3 kg/m^2^. Participants were more likely to be younger as BMI increased, and men had lower BMI compared with women. In addition, participants had lower proportion of current smokers, lower proportion of current alcohol drinkers, higher TC level, and higher TG level as BMI increased. During the average 9.8-year follow-up, 17,166 participants (9,503 men and 7,663 women) died. The baseline characteristics per each cohort are indicated in eTable 1.

**Table 1.** Clinical characteristics of all participants according to BMI category

BMI category	Number of participants	Sex men, *n* (%)	Age, years	BMI, kg/m^2^	Current smoker, *n* (%)	Current drinker, *n* (%)	SBP, mm Hg	DBP, mm Hg	TC, mg/dL	TG, mg/dL	Follow-up period, years	Follow-up period, person-years	Number of death	Crude death rates, per 1,000 person-years
<19	12,528	4,929 (39.3%)	61.1 (11.2)	17.9 (0.9)	3,711 (29.6%)	3,934 (31.7%)	128.0 (20.1)	75.0 (11.2)	190.2 (34.3)	82 (63–109)	9.7 (3.5)	121,054	2,243	18.5 (17.8–19.3)
19.0–19.9	12,156	4,886 (40.2%)	58.8 (11.0)	19.5 (0.3)	3,432 (28.2%)	4,247 (34.9%)	128.4 (18.9)	75.7 (11.0)	192.3 (35.5)	87 (65–119)	9.8 (3.3)	119,235	1,470	12.3 (11.7–13.0)
20.0–20.9	17,255	6,937 (40.2%)	58.3 (10.8)	20.5 (0.3)	4,667 (27.0%)	6,176 (36.1%)	129.6 (19.2)	76.5 (11.0)	194.2 (34.9)	93 (69–129)	9.9 (3.2)	170,979	1,854	10.8 (10.4–11.3)
21.0–21.9	21,259	8,454 (39.8%)	58.1 (10.6)	21.5 (0.3)	5,367 (25.2%)	7,690 (36.5%)	130.5 (18.7)	77.3 (10.9)	197.4 (35.6)	100 (74–141)	9.9 (3.1)	210,811	1,916	9.1 (8.7–9.5)
22.0–24.9	66,962	26,162 (39.2%)	58.3 (10.1)	23.4 (0.9)	14,779 (22.1%)	23,627 (35.5%)	133.1 (18.4)	79.1 (10.8)	202.5 (35.7)	117 (83–167)	9.9 (3.0)	661,424	5,593	8.5 (8.2–8.7)
25.0–25.9	16,253	6,153 (37.9%)	58.8 (9.8)	25.5 (0.3)	3,266 (20.1%)	5,539 (34.3%)	135.8 (17.9)	80.9 (10.7)	207.0 (35.6)	135 (97–192)	9.8 (2.9)	159,439	1,286	8.1 (7.6–8.5)
26.0–26.9	12,177	4,443 (36.5%)	58.9 (9.8)	26.5 (0.3)	2,312 (19.0%)	3,972 (32.9%)	137.0 (18.1)	81.9 (10.8)	208.0 (35.7)	141 (99–201)	9.9 (2.9)	120,071	986	8.2 (7.7–8.7)
27.0–27.9	8,163	2,749 (33.7%)	59.0 (9.7)	27.5 (0.3)	1,465 (17.9%)	2,524 (31.2%)	138.5 (17.9)	82.7 (10.7)	209.7 (35.7)	145 (103–209)	9.9 (2.9)	80,407	675	8.4 (7.8–9.1)
28.0–28.9	5,317	1,685 (31.7%)	59.1 (9.7)	28.5 (0.3)	911 (17.1%)	1,543 (29.3%)	139.6 (18.3)	83.6 (11.0)	210.0 (35.9)	151 (107–214)	9.9 (2.9)	52,451	431	8.2 (7.5–9.0)
29.0–29.9	3,277	880 (26.9%)	59.4 (9.7)	29.5 (0.3)	499 (15.2%)	836 (25.8%)	140.4 (18.0)	83.9 (10.8)	211.6 (35.4)	151 (109–207)	9.8 (2.9)	32,151	273	8.5 (7.5–9.6)
≥30.0	4,640	1,004 (21.6%)	58.5 (9.9)	31.9 (1.9)	695 (15.0%)	1,019 (22.2%)	142.5 (18.5)	85.6 (11.5)	214.0 (37.0)	155 (110–218)	9.8 (3.0)	45,459	439	9.7 (8.8–10.6)

Total	179,987	68,282 (38.2%)	58.7 (10.3)	23.3 (3.1)	41,104 (22.8%)	61,107 (34.2%)	133.1 (18.9)	79.0 (11.2)	201.3 (36.1)	113 (80–164)	9.9 (3.1)	1,773,481	17,166	9.7 (9.5–9.8)

BMI, body mass index; DBP, diastolic blood pressure; SBP, systolic blood pressure; TC, total cholesterol; TG, triglycerides.

Data are presented as mean (standard deviation), median (interquartile range) or as a number (%).

Follow-up period is presented as mean (standard deviation).

Crude death rates are presented with 95% confidence intervals.

**Figure 1.**
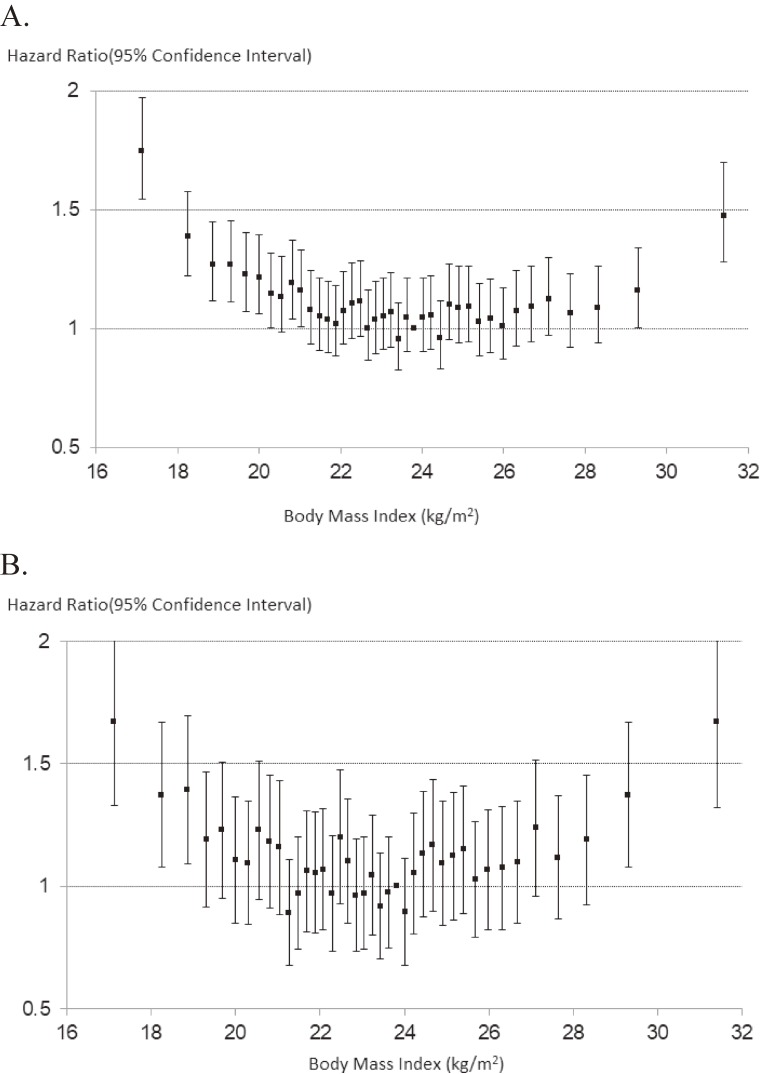
Relation between body mass index and all-cause mortality using 40 groups according to the quantile: EPOCH-JAPAN. A: Total participants, B: Healthy participants. Vertical line: hazard ratio for all-cause mortality; horizontal line: body mass index; dots showed age-sex-smoking adjusted hazard ratio and 95% confidence intervals. Study cohorts were used as stratification variable.

Figure 1A shows the age-, sex-, and smoking-adjusted relation between BMI and all-cause mortality for all participants. The U-shaped figure showed that significant increases in the risk of all-cause mortality were observed in those with BMI 21 kg/m^2^ or lower and BMI and 29 kg/m^2^ or higher. When we restricted the analyses to the healthy sample (ie, never smoking, total cholesterol 4.1 mmol/L or greater, and followed up for at least 5 years), significant increases in the all-cause mortality risks were still observed in subjects with BMI lower than 19 kg/m^2^ and 29 kg/m^2^ or higher (Figure 1B).

**Figure 2.**
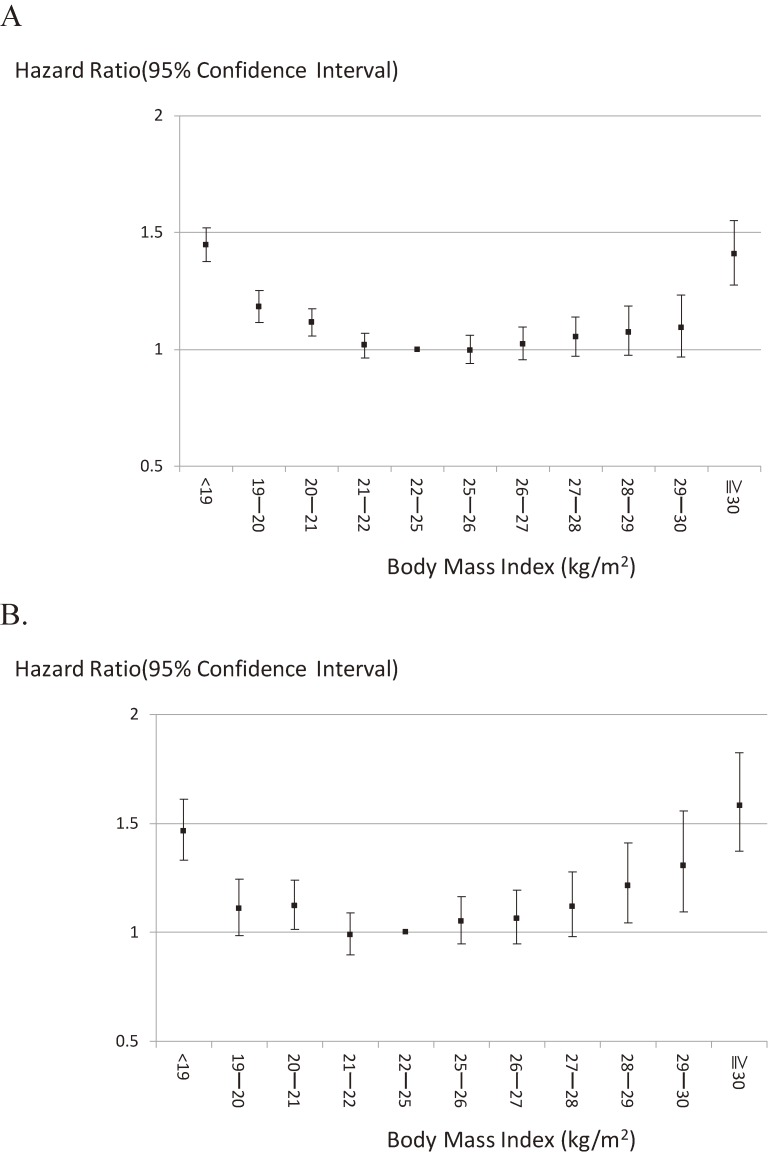
Relation between body mass index and all-cause mortality: EPOCH-JAPAN. A: Total participants, B: Healthy participants. Vertical line: hazard ratio for all-cause mortality; horizontal line: body mass index; dots showed age-sex-smoking adjusted hazard ratio and 95% confidence intervals. Study cohorts were used as stratification variable.

A U-shaped relationship was also obtained when a BMI range of 22–24.9 kg/m^2^ was used as the reference category (Figure 2A). Among all study participants, mortality rates were significantly higher in those with a BMI <19, 19–19.9, 20–20.9, or ≥30 kg/m^2^ compared with those in the reference category. Risk of BMI <19 kg/m^2^ was consistently higher in each cohort except for one cohort (Range of HRs, 0.83–1.82), and risk of BMI ≥30 kg/m^2^ was also consistently higher in each cohort except for one cohort (Range of HRs, 0.90–3.31). When we restricted to the healthy sample, increased mortality risk associated with BMI <21 kg/m^2^ was unchanged, but increased mortality risk associated with higher BMI became more evident. Statistically significantly increased mortality risk was observed from those with BMI 28 kg/m^2^ and more (Figure 2B).

**Figure 3.**
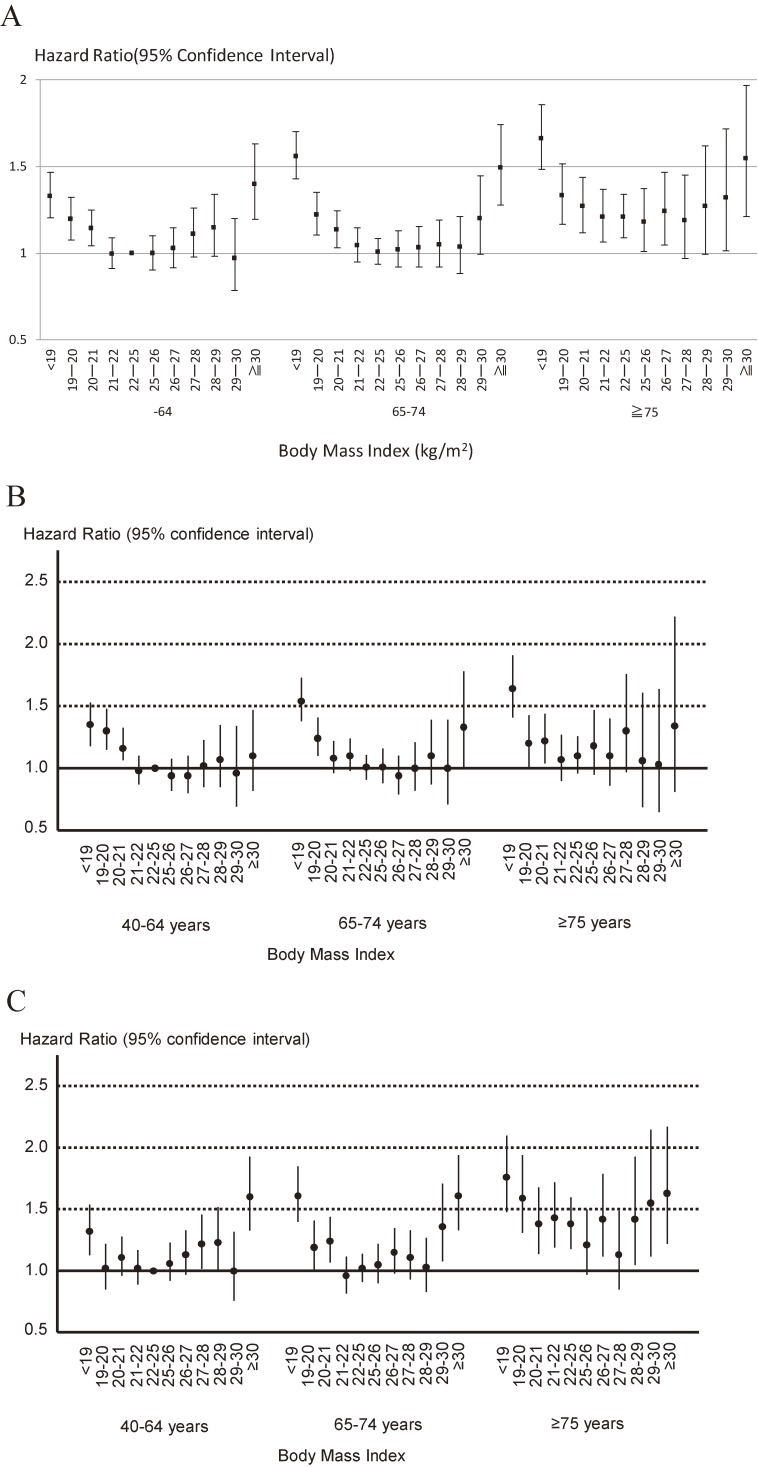
Relation between body mass index and all-cause mortality stratified by age category: EPOCH-JAPAN. A. Total participants, B. Only men, C. Only women. Vertical line: hazard ratio for all-cause mortality; horizontal line: body mass index; dots showed age-sex-smoking adjusted hazard ratio and 95% confidence intervals. Study cohorts were used as stratification variable. We treated 40–64 years old participants with BMI 22–25 kg/m^2^ as the reference group.

**Figure 4.**
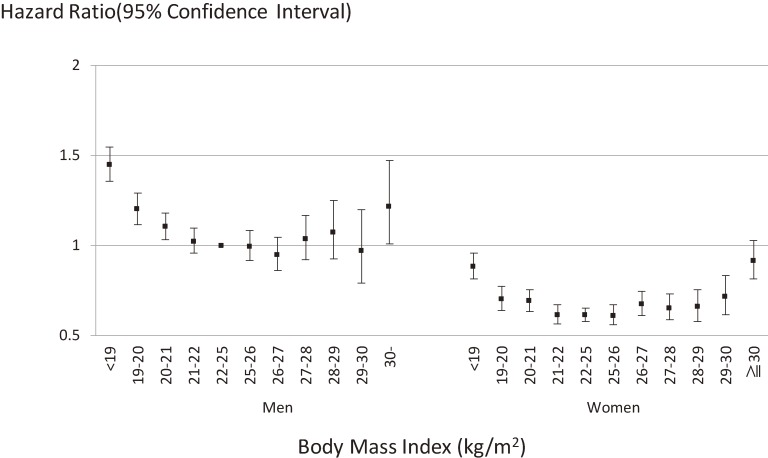
Relation between body mass index and all-cause mortality stratified by sex: EPOCH-JAPAN. Vertical line: hazard ratio for all-cause mortality; horizontal line: body mass index; dots showed age-smoking adjusted hazard ratio and 95% confidence intervals. Study cohorts were used as stratification variable. We treated men with BMI 22–25 kg/m^2^ as the reference group.

**Figure 5.**
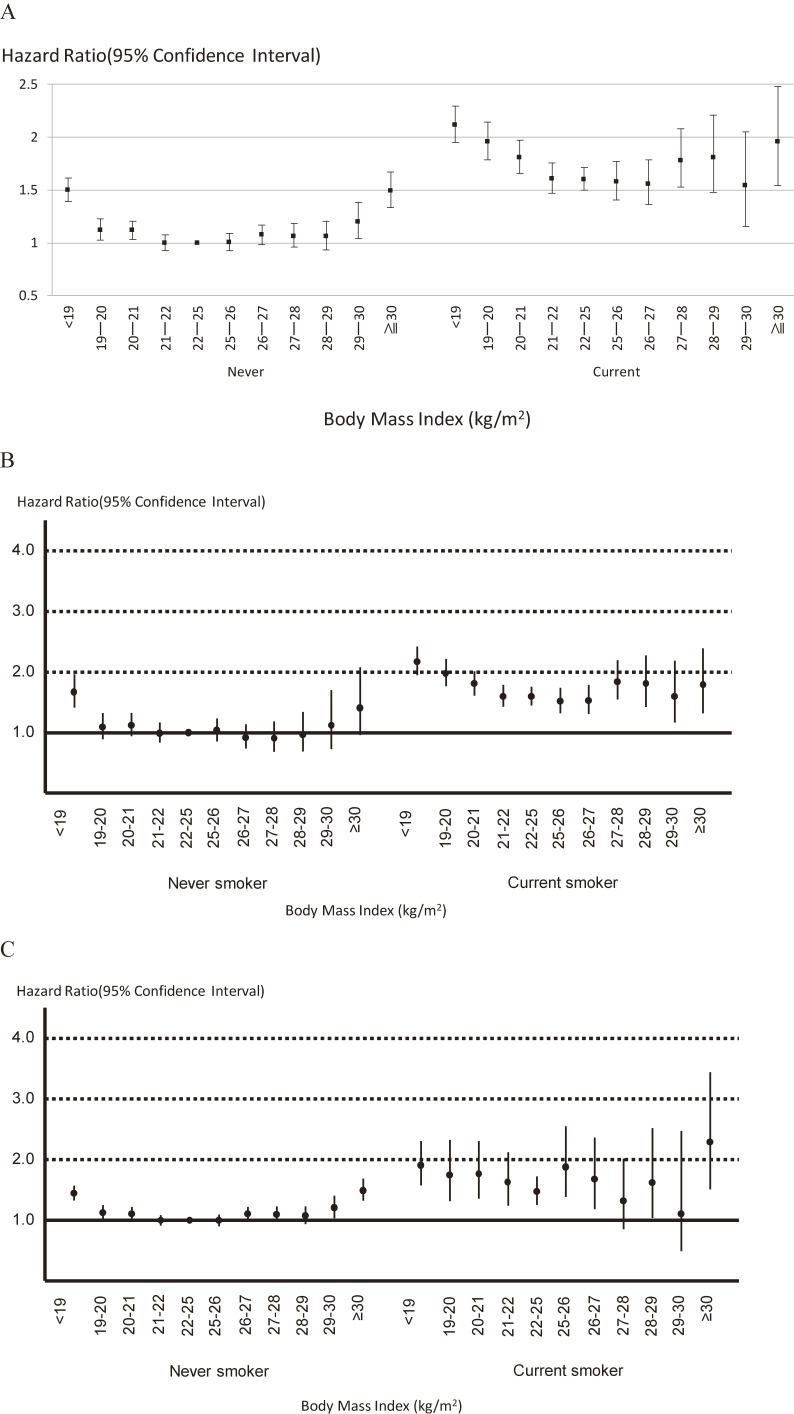
Relation between body mass index and all-cause mortality stratified by smoking status: EPOCH-JAPAN. A. Total participants, B. Only men, C. Only women. Vertical line: hazard ratio for all-cause mortality; horizontal line: body mass index; dots showed age-sex adjusted hazard ratio and 95% confidence intervals. We showed age-adjusted hazard ratio and 95% confidence intervals in sex-specific analyses. Study cohorts were used as stratification variable in all analyses. We treated never smokers with BMI 22–25 kg/m^2^ as the reference group.

Overall, age-stratified analyses showed that age did not affect the relation between BMI and mortality (*P* = 0.74) (Figure 3A). In sex-specific analyses, the impact of age on the association between BMI and all-cause death was essentially unchanged (Figure 3B for men and Figure 3C for women). This relation was also unaffected by sex (*P* = 0.32) (Figure 4) and smoking status (never smokers versus current smokers) (*P* = 0.12). However, it is noted that current smokers with a BMI of 22–24.9 kg/m^2^ (the lowest risk category for current smokers) were at greater risk of death than never smokers with a BMI <18.9 kg/m^2^ or ≥30 kg/m^2^ (the highest risk categories for never smokers) (Figure 5A). In sex-specific analyses, the impact of smoking status on the association between BMI and all-cause death was essentially unchanged (Figure 5B for men and Figure 5C for women).

## DISCUSSION

This large-scale prospective pooled analysis describes the relationship between BMI and all-cause mortality. It reconfirms that individuals with BMIs of 22.0–24.9 kg/m^2^ have the lowest mortality risk. These BMIs therefore represent the desired targets for reducing the risk of all-cause mortality in the Japanese population. No statistical interactions of age, sex, or smoking status with BMI for all-cause mortality were observed.

To identify favorable and unfavorable BMI ranges under no assumption of the shape of the relationship, 40 finely graduated groups were compared, and we found that mortality risks were significantly higher when the BMI was <22 kg/m^2^ and ≥30 kg/m^2^.

We also analyzed healthy subjects (never smoked, baseline TC level ≥4.1 mmol/L, followed up for at least 5 years) in our study. More pronounced increases in mortality risks were observed in healthy subjects with a BMI of 27–29 kg/m^2^ than in all subjects with a BMI of 27–29 kg/m^2^. This finding would be consistent with the previous finding that the relation between BMI and morality was modified via health condition, although interactions were not statistically significant in the present study.^4^ Individuals in poor health might have a lower BMI than healthy individuals. In our study, stratifying by mortality risk factors (age, sex, and smoking status) in individuals with a low BMI did not alter the relation between low BMI and increased mortality. Hence, a low BMI may be a mortality risk factor in and of itself. Further studies assessing the association between low BMI and all-cause mortality are needed to identify the underlying mechanisms.

In this study, we checked the presence of significant interactions of age, sex, and smoking with BMI for all-cause mortality, and we found that there were no evident interactions. This finding indicates that BMI may be a marker of all-cause mortality irrespective of age or sex.

In an analysis using never smokers with a BMI of 22–24.9 kg/m^2^ as the reference category, we found that current smokers with an ideal BMI (22–24.9 kg/m^2^) associated with lowest mortality had a higher mortality risk than did never smokers with a non-ideal BMI (<19 kg/m^2^ or ≥30 kg/m^2^). Fear for potential increases in weight or a subsequent incremental risk of metabolic syndrome, might deter some individuals from quitting smoking.^20^ However, our data clearly show that smoking prevention is an important priority.

The advantage of this study is its use of a large dataset and a relatively long follow-up period. Use of the EPOCH-JAPAN dataset allowed us to establish 40 groups for assessing the relation between BMI and mortality, as well as to explore in depth the interactions of BMI with age, sex, and smoking status.

The present study had several limitations. First, we note that the 13 prospective cohort studies in the dataset were performed many years before the pooled analysis (ie, the EPOCH-JAPAN study) was performed. Hence, it was difficult to obtain detailed information on diseases (eg, severe diabetes, respiratory disease, and cancer) that may have resulted in weight loss. It is possible that individuals with serious diseases were included in our analyses, even those presumably restricted to healthy individuals. Because serious illness might lead to death early in the follow-up period, excluding the data in the first 5 years of the follow-up from the analysis might compensate at least in part for the lack of disease-related information. Second, temporal change in BMI was not investigated because the BMI value was included at baseline data only in EPOCH-JAPAN study. Thus, upward or downward trends of BMI may induce misclassification of BMI categories. Finally, the measurement methods of covariates, including smoking status, were not identical among cohorts. We took into account the difference of measurement methods by considering cohort as strata in the Cox proportional hazard models.

In conclusion, we described the relation between BMI and all-cause mortality using data from a large pooled analysis of the Japanese population in the present study. A BMI of 22–24.9 kg/m^2^ correlated with a low risk of mortality, regardless of whether all participants or selected healthy participants were analyzed. Age, sex, and smoking status did not affect the relation between BMI and all-cause mortality. Regardless of their BMI, never smokers had a lower all-cause mortality risk than did current smokers with an ideal BMI; this finding emphasizes the urgency for effective anti-smoking programs.

## APPENDIX A. SUPPLEMENTARY DATA

eTable 1. Clinical characteristics of total participants according to each cohort
