# Supplementary material for: Association Between Body Mass Index and All-Cause Death in Japanese Population: Pooled Individual Participant Data Analysis of 13 Cohort Studies
Source: J Epidemiol. 2019 Dec 5;29(12):457–63. doi: 10.2188/jea.JE20180124 (PMC6859077; doi:10.2188/jea.JE20180124)
Supplement: Supplementary file 1 [file je-29-457-s001.pdf]

**eTable 1.** Clinical characteristics of total participants according to each cohort

| Cohort | Number of participants | Sex men, n (%) | Age, years | BMI, kg/m$^2$ | Current smoker, n (%) | Current drinker, n (%) | SBP, mm Hg | DBP, mm Hg | TC, mg/dL |
|---|---|---|---|---|---|---|---|---|---|
| Tanno-Sobetsu | 1,612 | 745 (46.2%) | 50.4 (6.8) | 23.6 (3.1) | 583 (36.2%) | 606 (37.7%) | 132.2 (19.6) | 82.1 (9.7) | 190.3 (38.0) |
| Ohsaki | 13,629 | 6,504 (47.7%) | 62.0 (9.6) | 23.9 (3.1) | 3,496 (25.7%) | 6,272 (47.7%) | 131.1 (17.6) | 78.6 (11.0) | 203.5 (35.1) |
| Ohasama | 2,330 | 928 (39.8%) | 59.1 (10.0) | 23.6 (3.1) | 500 (21.5%) | 652 (28.0%) | 131.1 (16.8) | 74.2 (10.9) | 197.8 (36.6) |
| Oyabe | 4,692 | 1,503 (32.0%) | 58.8 (9.8) | 23.0 (2.9) | 898 (19.1%) | 739 (15.8%) | 127.8 (20.0) | 76.1 (11.2) | 195.7 (36.5) |
| YKK | 4,872 | 3,167 (65.0%) | 50.7 (6.4) | 22.4 (2.7) | 1,889 (38.8%) | 2,970 (61.0%) | 118.6 (15.1) | 72.6 (11.8) | 197.5 (35.2) |
| SPMI | 4,495 | 1,932 (43.0%) | 54.2 (8.1) | 22.8 (2.9) | 1,247 (27.7%) | 2,039 (45.5%) | 132.7 (17.6) | 80.2 (10.5) | 196.7 (35.5) |
| Suita | 4,844 | 2,301 (47.5%) | 58.8 (10.7) | 22.6 (3.1) | 1,385 (28.6%) | 2,466 (51.1%) | 129.5 (21.6) | 78.4 (12.0) | 210.6 (37.0) |
| RERF | 4,203 | 1,312 (31.2%) | 61.0 (11.5) | 22.7 (3.5) | 1,006 (23.9%) | 1,895 (49.8%) | 134.1 (22.4) | 82.4 (11.9) | 211.7 (39.2) |
| Hisayama | 2,631 | 1,113 (42.3%) | 59.0 (11.7) | 22.8 (3.2) | 661 (25.1%) | 806 (30.7%) | 133.7 (21.3) | 77.8 (11.3) | 206.7 (42.2) |
| JACC | 27,479 | 10,147 (36.9%) | 57.3 (9.7) | 23.1 (3.1) | 5,973 (21.7%) | 10,659 (39.4%) | 133.3 (19.4) | 79.2 (11.3) | 197.9 (36.8) |
| NIPPON DATA80 | 7,157 | 3,147 (44.0%) | 56.2 (10.9) | 22.8 (3.2) | 2,271 (31.7%) | 2,983 (41.7%) | 140.3 (21.8) | 83.0 (12.2) | 191.8 (34.1) |
| NIPPON DATA90 | 6,013 | 2,572 (42.8%) | 57.3 (11.5) | 23.1 (3.2) | 1,651 (27.5%) | 1,703 (28.3%) | 139.0 (20.5) | 82.8 (11.8) | 206.9 (38.5) |
| IPHS | 96,030 | 32,911 (34.3%) | 59.5 (10.3) | 23.5 (3.2) | 19,544 (20.4%) | 27,317 (28.4%) | 133.7 (17.9) | 78.9 (10.7) | 202.3 (35.2) |
| Total | 179,987 | 68,282 (37.9%) | 58.7 (10.3) | 23.3 (3.1) | 41,104 (22.8%) | 61,107 (34.2%) | 133.1 (18.9) | 79.0 (11.2) | 201.3 (36.1) |

eTable 1 (continued).

| Cohort | TG, mg/dL | Follow-up period, years | Follow-up period, py | Number of deaths | Crude death rate, per 1,000 py |
|---|---|---|---|---|---|
| Tanno-Sobetsu | 95 (72-132) | 18.6 (3.6) | 30,061 | 151 | 5.0 (4.3-5.9) |
| Ohsaki | 108 (75-161) | 6.0 (1.4) | 81,929 | 708 | 8.6 (8.0-9.3) |
| Ohasama | 115 (82-167) | 10.3 (2.5) | 24,052 | 317 | 13.2 (11.8-14.7) |
| Oyabe | 111 (81-157) | 9.9 (1.7) | 46,640 | 536 | 11.5 (10.6-12.5) |
| YKK | 89 (64-124) | 11.1 (2.3) | 53,867 | 89 | 1.7 (1.3-2.0) |
| SPMI | 109 (77-156) | 9.2 (2.9) | 41,189 | 211 | 5.1 (4.5-5.9) |
| Suita | 104 (75-150) | 5.8 (1.8) | 28,070 | 248 | 8.8 (7.8-10.0) |
| RERF | 122 (89-174) | 14.8 (4.3) | 62,009 | 1,508 | 24.3 (23.1-25.6) |
| Hisayama | 97 (70-141) | 10.7 (2.9) | 28,145 | 336 | 11.9 (10.7-13.3) |
| JACC | 101 (73-144) | 9.5 (2.0) | 261,727 | 2,218 | 8.5 (8.1-8.8) |
| NIPPON DATA80 | N/A | 16.7 (4.5) | 119,736 | 1,937 | 16.2 (15.5-16.9) |
| NIPPON DATA90 | 112 (79-165) | 9.5 (1.7) | 56,910 | 697 | 12.2 (11.4-13.2) |
| IPHS | 118 (83-171) | 9.8 (1.8) | 939,146 | 8,210 | 8.7 (8.6-8.9) |
| Total | 113 (80-164) | 9.9 (3.1) | 1,773,481 | 17,166 | 9.7 (9.5-9.8) |

BMI, body mass index; DBP, diastolic blood pressure; N/A, not assessed; py, person-years; SBP, systolic blood pressure; TC, total cholesterol; TG, Triglycerides.

Data are presented as mean (standard deviation), median (interquartile range) or as a number.

Crude death rates are presented with 95% confidence intervals.
